# Total marrow irradiation versus total body irradiation using intensity-modulated helical tomotherapy

**DOI:** 10.1007/s00432-022-04565-2

**Published:** 2023-01-06

**Authors:** Mümtaz Köksal, Laura Kersting, Felix Schoroth, Stephan Garbe, David Koch, Davide Scafa, Gustavo R. Sarria, Christina Leitzen, Annkristin Heine, Tobias Holderried, Peter Brossart, Eleni Zoga, Ulrike Attenberger, Leonard C. Schmeel

**Affiliations:** 1 Department of Radiation Oncology, University Hospital Bonn, Bonn, Germany; 2 Department of Internal Medicine—Oncology/ Hematology and Rheumatology, University Hospital Bonn, Bonn, Germany; 3 Deprtment of Radiation Oncology, Sana Hospital Offenbach, Offenbach, Germany; 4 Department of Radiology, University Hospital Bonn, Bonn, Germany

**Keywords:** Hematological malignancy, Hematopoietic stem cell transplantation, Radiotherapy, Total body irradiation, Total marrow irradiation, Treatment planning

## Abstract

**Background:**

Total body irradiation (TBI) is often a component of the conditioning regimen prior to hematopoietic stem cell transplantation in patients with hematological malignancies. However, total marrow irradiation (TMI) could be an alternative method for reducing radiation therapy-associated toxicity, as it specifically targets the skeleton and thus could better protect organs at risk. Here, we compared dosimetric changes in irradiation received by the target volume and organs at risk between TBI and TMI plans.

**Materials and methods:**

Theoretical TMI plans were calculated for 35 patients with various hematological malignancies who had already received TBI in our clinic. We then statistically compared irradiation doses between the new TMI plans and existing TBI plans. We examined whether TMI provides greater protection of organs at risk while maintaining the prescribed dose in the targeted skeletal area. We also compared beam-on times between TBI and TMI.

**Results:**

TMI planning achieved significant reductions in the mean, minimum, and maximum irradiation doses in the lungs, kidneys, liver, spleen, and body (i.e., remaining tissue except organs and skeleton). In particular, the mean dose was reduced by 49% in the liver and spleen and by 55–59% in the kidneys. Moreover, TMI planning reduced the corpus beam-on time by an average of 217 s.

**Conclusion:**

TMI planning achieved significant dose reduction in organs at risk while still achieving the prescribed dose in the target volume. Additionally, TMI planning reduced the beam-on time for corpus plans despite a high modulation factor.

**Graphical abstract:**

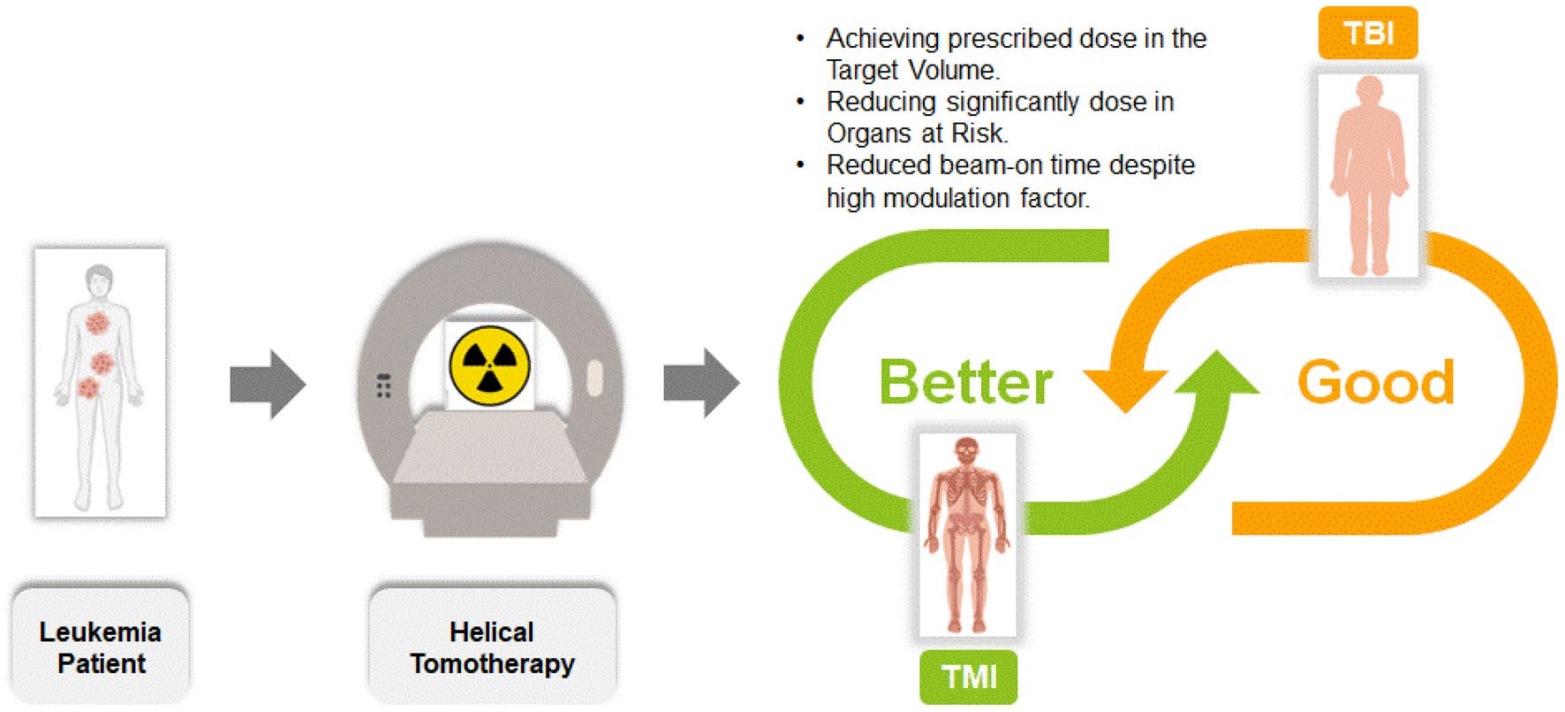

**Supplementary Information:**

The online version contains supplementary material available at 10.1007/s00432-022-04565-2.

## Introduction

Hematological malignancies, such as acute lymphoblastic leukemia and acute myeloid leukemia, can be treated with hematopoietic stem cell transplantation (HSCT) (Snowden et al. 2022). Before HSCT, a myeloablative conditioning regimen is performed to eradicate tumor cells and achieve immunosuppression for better acceptance of the donor transplant (Vriesendorp 2003). Part of this conditioning regimen involves the combination of chemotherapy and irradiation, which achieves better overall survival and a lower relapse rate (Peters et al. 2021). A widely used and effective method of irradiation is total body irradiation (TBI), which involves irradiation of the entire body (Wills et al. 2016). However, dose escalation in TBI treatments is limited by increasing toxicity due to higher levels of radiation received by organs at risk (OARs). Although the probability of relapse could be reduced through dose escalation, this entails a risk of mortality caused by toxicity (Clift et al. 1990; Clift et al. 1991).

In our clinic, TBI is carried out using helical tomotherapy (TomoTherapy^®^, Accuray, WI, USA), in which the radiation source rotates helically around the table, while it moves through the gantry opening. Prior to treatment, a megavoltage computed tomography (MVCT) scan is obtained, which must match the already existing planning kilovoltage computed tomography scan to ensure accurate patient alignment. Due to the limitation of helical tomotherapy to a length of 135 cm, irradiation is carried out separately for the corpus and legs, with the patient first treated head-first and then feet-first.

An alternative method to TBI is total marrow irradiation (TMI) (Haraldsson et al. 2019), in which intensity-modulated and precise irradiation of the entire skeleton (i.e., planning target volume) including the hematopoietic bone marrow is performed while sparing OARs from radiation (Hui et al. 2005). This method could reduce toxicity in OARs and thus allow dose escalation in the planning target volume (Hui et al. 2017; Wong et al. 2013). Like TBI, TMI is also carried out using helical tomotherapy.

In the present study, we calculated theoretical TMI plans for 35 patients with hematological malignancies who underwent TBI to compare the two methods in terms of their doses and beam-on time. This theoretical approach would not be feasible for the same patients in vivo and avoids inaccuracies caused by matching patients in different treatment groups. To our knowledge, this is the first investigation of the exact irradiation of the entire skeleton down to the extremities, including the fingers and toes.

## Materials and methods

### Patients

All 35 patients who received TBI due to hematological malignancy between 2013 and 2022 at our university medical center were retrospectively included in this study. Of these, 19 patients were female and 16 were male (Table 1). Mean age was 46.2 years at the time of inpatient admission for irradiation (median, 49.3 years). Age ranged between 13 and 72 years, with four patients under 18 years of age.

**Table 1:** Patient characteristics and diagnoses

Patient characteristics	
*n*	35
Gender	
Male	16
Female	19
Age in years	
Median	49.3
Mean	46.2
0–17	4
18–30	3
31–40	6
41–50	8
51–60	5
61–75	9

Diagnosis	
AML	12
ALL, Common-B	10
ALL, Pre-T	3
MDS	2
ALL, Pre-B	1
ALL, Pro-B	1
ALL, T NOS	1
Burkitt-ALL	1
Mast cell leukemia	1
MPAL	1
MPN	1
CMML	1
DLBCL	1

*AML* acute myeloid leukemia, *ALL* acute lymphoblastic leukemia, *MDS* myelodysplastic syndrome, *MPAL* mixed-phenotype acute leukemia, *MPN* myeloproliferative neoplasms, *CMML* chronic myelomonocytic leukemia, *DLBCL* diffuse large B-cell lymphoma

Patients with various hematological malignancies were included. Acute lymphoblastic leukemia was the most common diagnosis (*n* = 16 patients), followed by acute myeloid leukemia (*n* = 12 patients). Two patients had two diagnoses at the same time (Supplemental Table 1).

European Group for Blood and Marrow Transplantation and Sorror scores were available for adult patients only (Supplemental Table 1). The European Group for Blood and Marrow Transplantation score describes the individual probability for HSCT success, whereas the Sorror score describes comorbidities prior to HSCT. Data on the line of therapy were also available. Most patients (*n* = 24) received first-line therapy, with fewer patients receiving second-line (*n* = 9) or third-line (*n* = 2) therapy.

**Fig. 1:**
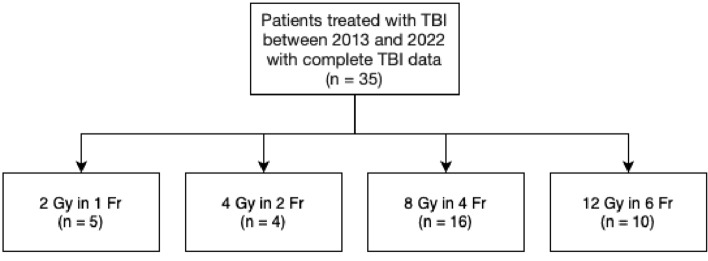
Patient selection

The prescribed radiation dose for TBI varied among patients. Ten patients received 12 Gy in six fractions, 16 patients received 8 Gy in four fractions, 4 patients received 4 Gy in two fractions, and 5 patients received 2 Gy in one fraction (Fig. 1). The radiation dose in the following OARs was compared for all patients: eyes, lenses, lungs, kidneys, liver, spleen, and body (i.e., remaining tissue except organs and skeleton). One participant had a splenectomy.

### Treatment planning

For treatment planning, we first contoured the entire skeleton in pre-existing TBI computed tomography (CT) scans (corpus and legs) using Aria^®^ (ARIA Oncology Information System, Varian Medical Systems, CA, USA). All distal extremities, including fingers and toes, were included using a contouring assistant utilizing Hounsfield units for bone recognition. Due to imprecisions in the contouring assistant, manual post-processing was required for every layer of the CT, which took ~ 4 h per patient. In addition, we contoured OARs that should be spared from radiation.

Next, corpus and leg CT scans with contoured volumes were transferred to Tomotherapy^®^ HiART II planning software to calculate theoretical TMI plans. For this purpose, the prescribed dose used for TBI was selected as the total dose for each patient. In addition, the following parameters were used for TMI planning: field width of 5.1 cm, pitch of 0.4, and modulation factor (MF) of 2.6. Field width and pitch were selected according to institutional protocols and were the same as those used for TBI. We chose 2.6 as the MF for TMI planning to achieve a high degree of conformity in the target volume, whereas an MF of 2 was used for corpus plans and an MF of 1.6 for leg plans in TBI.

The skeleton was selected as the target volume (category “Target Objectives”), and OARs were selected in the category “Critical Constraints”. In leg plans, only the body volume was spared. The calculations were performed in < 200 iterations, adjusting the penalty points every 15–20 iterations. The last calculation was performed with high resolution. The calculated doses for the corpus and legs were reimported into Aria^®^ and displayed as dose-volume histograms (DVHs). “Corpus-plan sums” were calculated for the skeleton and body volume. TMI and corresponding TBI data were exported for statistical analysis. In addition, we compared beam-on times between TBI and TMI separately for corpus and leg plans.

### Variables

D_mean_ was the average dose received in the respective organ. D_2_ was the minimum dose received by 2% of the volume, representing the maximum dose (Kataria et al. 2012). D_98_ was the minimum dose received by 98% of the volume, representing the minimum dose (Kataria et al. 2012). D_p_ was the prescribed dose. The beam-on time (in s) was measured separately for corpus and leg plans due to the limitation of helical tomotherapy to 135 cm. The homogeneity index (HI) describes the uniformity of dose distribution in the target volume. We used formula (1) to calculate the HI, with a lower value indicating a more homogeneous dose in the target volume; an ideal value was 0 (Kataria et al. 2012)

1 $$HI=\frac{(D2-D98)\times{100}}{Dp}.$$

### Statistical analysis

For descriptive statistics, we divided patients into 2, 4, 8, and 12 Gy subgroups according to their D_p_. Differences in D_mean_, D_2_, and D_98_ between TMI and TBI plans and corresponding mean values were calculated for each organ in each Gy subgroup. We also calculated relative deviation for the entire patient cohort by calculating TMI–TBI differences for D_mean_, D_2_, and D_98_ for each organ and dividing these differences by the D_mean_, D_2_, and D_98_ of the TBI plans. Mean and median values and standard deviations of all relative deviations were calculated for each organ without considering Gy subgroup. All calculations were also performed for the target volume (i.e., skeleton) to verify that the originally prescribed TBI dose could be achieved by TMI. Furthermore, we calculated the HI for the skeleton from the TMI data.

We also compared beam-on times between TBI and TMI by calculating mean and median values and standard deviations separately for corpus and leg plans.

Statistical analysis was performed using Microsoft^®^ Excel (version: 16.66.1, Microsoft, WA, USA) and IBM^®^ SPSS^®^ Statistics (version: 28.0.1.1 (14), IBM, NY, USA). Wilcoxon signed-rank tests were used for pairwise comparisons of continuous variables.

## Results

**Fig. 2:**
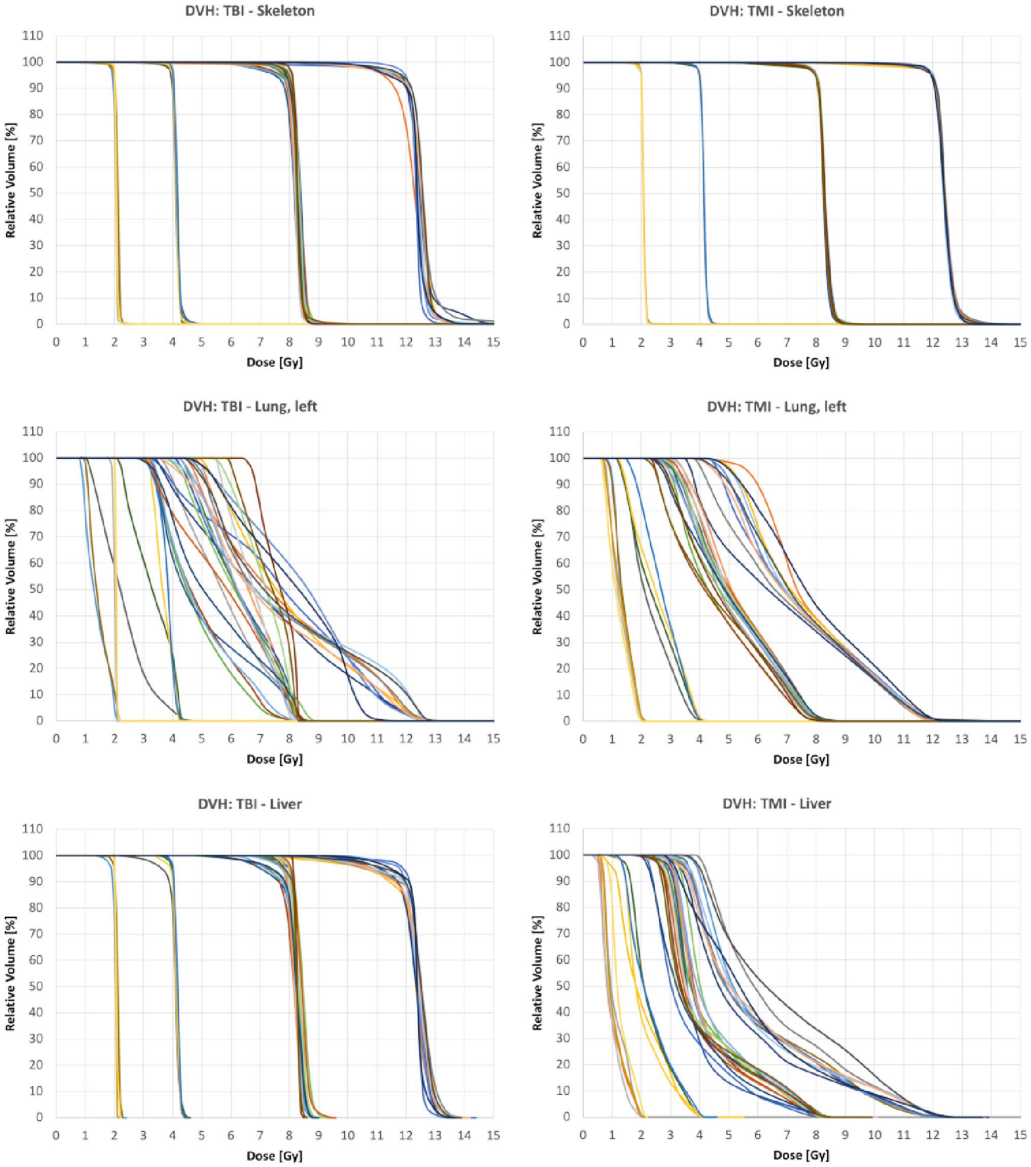
Comparison of DVHs between TBI and TMI for the skeleton, left lung, and liver for all patients. The clustering of data corresponding to different Gy subgroups (2, 4, 8, and 12 Gy)

DVHs for the skeleton, left lung, and liver for all patients show that we achieved the D_p_ for the skeleton more consistently with TMI than with TBI (Fig. 2). Notably, the lung, which was spared in most TBI plans, showed more consistent sparing with TMI planning. Also, TMI planning achieved marked improvements in dose sparing of the liver, which was not spared in TBI.

### Dose in OARs

For the liver, the D_mean_ was 3% above the D_p_ in TBI but was reduced by 49.32% through TMI planning (Table 2b). The D_2_ and D_98_ were 7–10% above and 3–15% below the D_p_ in TBI but were reduced by 10.39% and 65.95%, respectively, through TMI planning.

**Table 2:** (a) Average total reduction in D_mean_, D_2_, and D_98_ for each organ and Gy subgroup and (b) mean relative reduction and standard deviation (SD) for all patients

(a) Average total deviation												
	D_mean_ deviation				D_2_ deviation				D_98_ deviation			
	2 Gy	4 Gy	8 Gy	12 Gy	2 Gy	4 Gy	8 Gy	12 Gy	2 Gy	4 Gy	8 Gy	12 Gy
Eye, l	0*.*32	0*.*65	0*.*18	0*.*36	0*.*23	0*.*12	−0*.*31	− 0*.*70	0*.*30	0*.*63	0*.*25	0*.*56
Eye, r	0*.*32	0*.*74	0*.*36	0*.*33	0*.*14	0*.*21	0*.*05	− 0*.*83	0*.*34	0*.*65	0*.*28	0*.*65
Lens, l	0*.*23	0*.*66	0*.*21	0*.*34	0*.*19	0*.*73	0*.*13	0*.*04	0*.*25	0*.*63	0*.*27	0*.*54
Lens, r	0*.*29	0*.*67	0*.*26	0*.*41	0*.*25	0*.*72	0*.*23	0*.*26	0*.*34	0*.*67	0*.*27	0*.*67
Lung, l	0*.*49	0*.*76	1*.*03	0*.*33	0*.*13	0*.*31	0*.*30	0*.*48	0*.*77	1*.*06	1*.*43	− 0*.*10
Lung, r	0*.*50	0*.*75	1*.*11	0*.*28	0*.*12	0*.*21	0*.*26	0*.*51	0*.*77	0*.*91	1*.*31	− 0*.*17
Kidney, l	1*.*10	1*.*78	3*.*89	3*.*57	0*.*56	1*.*04	2*.*74	1*.*29	1*.*15	1*.*62	3*.*54	3*.*06
Kidney, r	1*.*16	1*.*76	3*.*89	4*.*02	0*.*62	1*.*33	3*.*09	3*.*02	1*.*19	1*.*63	3*.*46	3*.*23
Spleen	1*.*05	1*.*94	3*.*91	6*.*16	0*.*31	0*.*57	1*.*11	2*.*14	1*.*36	2*.*70	4*.*75	7*.*01
Liver	0*.*99	1*.*94	4*.*07	6*.*26	0*.*21	0*.*47	0*.*81	1*.*63	1*.*32	2*.*49	4*.*76	6*.*75
Body	0*.*57	1*.*18	2*.*46	3*.*51	0*.*10	0*.*21	0*.*29	0*.*72	1*.*09	1*.*76	3*.*38	3*.*10
Skeleton	− 0*.*01	− 0*.*01	− 0*.*04	0*.*01	− 0*.*03	0*.*01	− 0*.*07	0*.*25	− 0*.*01	− 0*.*02	− 0*.*26	− 0*.*55

(b) Mean relative deviation						
	D_mean_	SD	D_2_	SD	D_98_	SD
Eye, l	− 17*.*33	67*.*70	− 23*.*08	63*.*29	− 16*.*38	58*.*67
Eye, r	14*.*48	66*.*65	− 23*.*88	69*.*47	− 10*.*78	51*.*67
Lens, l	25*.*04	65*.*80	− 31*.*80	74*.*10	− 18*.*34	59*.*27
Lens, r	− 18*.*31	60*.*75	− 23*.*47	72*.*71	− 10*.*38	51*.*34
Lung, l	14*.*12	13*.*67	4*.*46	3*.*83	22*.*01	26*.*44
Lung, r	14*.*62	14*.*90	3*.*95	3*.*44	19*.*31	29*.*88
Kidney, l	55*.*46	13*.*14	26*.*65	19*.*58	60*.*13	15*.*66
Kidney, r	58*.*60	12*.*27	35*.*19	14*.*65	61*.*96	15*.*15
Spleen	49*.*12	4*.*70	14*.*07	4*.*01	68*.*19	5*.*26
Liver	49*.*32	3*.*70	10*.*39	2*.*82	65*.*95	4*.*98
Body	29*.*36	2*.*57	4*.*02	3*.*41	59*.*04	11*.*21
Skeleton	− 0*.*33	1*.*13	− 0*.*06	2*.*24	− 3*.*36	7*.*33

**Table 3:** Wilcoxon signed-rank test results for dose reductions achieved through TMI planning

	*p* value		
	D_mean_	D_2_	D_98_
Eye, left	0*.*731	0*.*555	0*.*670
Eye, right	0*.*502	0*.*731	0*.*922
Lens, left	0*.*334	0*.*310	0*.*432
Lens, right	0*.*635	0*.*819	0*.*635
Lung, left	*< *0*.*001	*< *0*.*001	*< *0*.*001
Lung, right	*< *0*.*001	*< *0*.*001	*< *0*.*001
Kidney, left	*< *0*.*001	*< *0*.*001	*< *0*.*001
Kidney, right	*< *0*.*001	*< *0*.*001	*< *0*.*001
Spleen	*< *0*.*001	*< *0*.*001	*< *0*.*001
Liver	*< *0*.*001	*< *0*.*001	*< *0*.*001
Body	*< *0*.*001	*< *0*.*001	*< *0*.*001
Skeleton	0*.*310	0*.*743	0*.*001

For the spleen, the D_mean_ was 2–3% above the D_p_ in TBI but was reduced by 49.12% through TMI planning. The D_2_ and D_98_ were 7–11% above and 5–16% below the D_p_ in TBI but were reduced by 14.07% and 68.19%, respectively, through TMI planning.

For the kidneys, the D_mean_ was 11–37% below the D_p_ in TBI but was reduced by 55.46% for the left kidney and 58.60% for the right kidney through TMI planning. The D_2_ and D_98_ were 12% above and 19–54% below the D_p_ in TBI but were reduced by 26.65% and 60.13% for the left kidney and 35.19% and 61.96% for the right kidney, respectively, through TMI planning.

For the lungs, the D_mean_ was 11–37% above the D_p_ in TBI but was reduced by 14.12% for the left lung and 14.62% for the right lung through TMI planning. The D_2_ and D_98_ were 1–5% above and 22–65% below the D_p_ in TBI but were reduced by 4.46% and 22.01% for the left lung and 3.95% and 19.31% for the right lung, respectively, through TMI planning.

For the body (i.e., remaining tissue except organs and skeleton), the D_mean_ was 1–3% above the D_p_ in TBI but was reduced by 29.36% through TMI planning. The D_2_ and D_98_ were 11–14% above and 19–50% below the D_p_ in TBI but were reduced by 4.02% and 59.04%, respectively, through TMI planning.

For the eyes and lenses, TMI planning led to increased doses (Table 2b). However, the doses had high standard deviations of ~ 60% arising from inconsistent TBI planning values​, in part due to the small volumes of these organs. However, because the mean values were similar between TBI and TMI within the Gy subgroups (Supplemental Table 2), there does not appear to be any major change in the total deviations (Table 2a) for the Gy subgroups.

Wilcoxon signed-rank tests showed significant dose reductions (p < 0.001) for all organs except the eyes and lenses (Table 3).

### Dose in the target volume

In the skeleton, we were able to achieve or even slightly exceed the D_p_ through TMI planning. The TBI D_mean_ was 3% above the D_p_, which was increased by 0.33% through TMI planning. The TBI D_2_ was 8–11% above the D_p_, which was increased by 0.06% through TMI planning. The largest increase was for D_98_, which was 3–11% below the D_p_ in TBI but was increased by 3.36% through TMI planning. Wilcoxon signed-rank tests showed no significant change for D_mean_ or D_2_ but a significant increase for D_98_ (*p* = 0.001, Table 3).

The mean HI for the skeleton was 13.21 with TMI planning, with a minimum value of 9.54 and maximum value of 38.51 (Table 4). However, this maximum value was an outlier, as the second highest value was 16.2.

**Table 4:** HI for the skeleton (TMI only)

	Homogeneity index $HI = \frac{{(D_{2} - D_{{98}} )}}{{D_{p} }}*100$
Mean	13.21
Median	12.25
SD	4.67
Min	9.54
Max	38.51

### Beam-on time

As beam-on times were only available for 19 patients undergoing TBI, only these patients were used for comparisons between TBI and TMI. For the corpus, TMI planning reduced mean beam-on time by 217 s (median, 248 s) (Table 5). For the legs, TMI planning increased mean beam-on time by 148 s (median, 208 s).

**Table 5:** Comparison of beam-on time between TBI and TMI plans

	TMI (*n* = 19)		TBI (*n* = 19)	
	Corpus	Legs	Corpus	Legs
Mean [s]	1031*.*2	772*.*4	1238*.*2	624*.*9
Median [s]	1027*.*2	785*.*7	1285*.*3	577*.*4
SD [s]	83*.*8	78*.*9	163*.*6	142*.*9

## Discussion

We found that the radiotherapy dose in OARs could be significantly reduced through TMI planning compared with TBI planning. To our knowledge, this is the first study to statistically compare TBI and TMI plans for the same patient group with various diagnoses. The advantage of this approach is that patient characteristics as well as OARs are identical between planning approaches, which avoids inaccuracies caused by matching separate groups of patients.

TMI targets and obliterates the hematopoietic bone marrow, where tumor cells are formed in hematological malignancies. Thus, whereas chemotherapy kills tumor cells that have already been formed and are circulating in blood vessels or organs, TMI prevents the formation of new tumor cells. However, there is a risk that tumor cells that are still circulating after TMI could lead to organ infiltration or an increased extramedullary relapse rate. Kim et al. showed that extramedullary relapse rates are similar after TMI (or total marrow and lymphoid irradiation) and TBI (Kim et al. 2014). Therefore, although TBI and TMI are different approaches, they can be regarded as equivalent in this respect.

We found that TMI achieved particularly high-dose reduction in the liver, spleen, and kidneys. Its effect on the lungs, however, was smaller, because lung shielding was already included in most TBI plans (Wilhelm-Buchstab et al. 2020). However, Hui et al. and Anderson et al. showed that conventional lung blocks result in a higher risk of relapse, because, among other reasons, the ribs and sternum cannot be adequately irradiated (Hui et al. 2004; Anderson et al. 2001; Ellis 1961). This relapse rate could possibly be reduced through TMI, as both the ribs and sternum are part of the target volume.

Although the relative dose reduction was in a similar range for respective organs across all Gy subgroups (e.g., D_mean_ of 49.32 ± 3.7% for the liver, Table 2b), the 12 Gy group had the largest total dose reduction. TMI planning may thus be particularly useful for higher irradiation doses, especially for dose escalations higher than 12 Gy. Indeed, Hui et al. described TMI dose escalation up to 15 Gy (Hui et al. 2017).

A disadvantage of TMI planning is its addition to the daily clinical practice workload, as the contouring of OARs and target volume took up to 4 h per patient. At our clinic, not many patients receive this type of treatment, therefore the effort seems reasonable. Moreover, time savings and greater precision could be achieved using deep learning or atlas-based auto-contouring of the OARs and target volume. For instance, deep learning software such as Limbus Contour® (Limbus AI, SK, Canada) show promising results (Wong et al. 2020).

TMI planning reduced the beam-on time for corpus plans by an average of 217 s. This reduction was achieved despite an MF ($MF=\frac{maximum\, leaf-open\, time}{average\, leaf-open\, time}$) (Fenwick et al. 2004) of 2.6, which was higher than the MF of the TBI plans (corpus MF = 2). Higher MFs directly result in longer beam-on times (Hui et al. 2005) but also increase conformity. As the skeletal target volume requires a high level of conformity in TMI compared with TBI, we chose a high MF of 2.6. The targeted dose application in TMI (i.e., higher MF) would therefore suggest a longer beam-on time. However, we achieved a slight reduction in the beam-on time in the corpus plans. One reason for this reduction in beam-on time could be due to leaf movement. If leaf movement blocks the dose outside the target volume (i.e., conformity), there is no effect on irradiation time. If leaf movement modulates the dose within the target volume (i.e., homogeneity), the dose rates will be reduced, and the irradiation time will be longer. This could impact the beam-on time in TMI, as less modulation is required within the target volume. Another reason for the reduced TMI corpus beam-on time could be that helical tomotherapy does not have a flattening filter, which means that the same dose can be delivered in less time, as the target volume is limited in TMI. Therefore, despite a higher MF with TMI planning, corpus irradiation times were not increased, which could be another advantage of TMI and have a positive effect on patient acceptance.

Considering the leg plans, TMI planning increased the beam-on time by an average of 148 s. This increase could be because the legs were irradiated with static fields (i.e., complete leaf opening) in TBI, leading to shorter irradiation times. By contrast, in TMI, the legs are irradiated helically with non-static fields (i.e., dose modulation by leaf movement), which is more time-consuming. The only slight lengthening of irradiation time for this more complex method is therefore an acceptable finding. Another option for reducing the overall treatment time is to use optical surface scanning instead of an MVCT scan to align the patient prior to irradiation. Indeed, as described by Haraldsson et al., the legs can be positioned faster with optical surface scanning than with an MVCT scan (Haraldsson et al. 2019).

In addition to our findings concerning dose reduction and beam-on time, Haraldsson et al. describe further advantages of TMI in terms of engraftment, graft-versus-host-disease, and relapse-free survival, which further support the benefits of TMI (Haraldsson et al. 2021).

## Conclusions

Our study shows that the use of TMI instead of TBI in the conditioning regimen prior to HSCT allows a significant radiation dose reduction in OARs. The use of TMI planning reduced the D_mean_ by 49% in the liver and spleen and 55–59% in the kidneys while still achieving the D_p_ in the target volume (i.e., the skeleton). In addition, the HI indicated a uniform dose distribution in the target volume, and the beam-on time for the corpus plans was reduced despite a higher MF.

## Supplementary Information

Below is the link to the electronic supplementary material.

Supplementary file1 (PDF 242 KB)

## Data Availability

All data relevant to this publication have been included into the manuscript’s body.

## Abbreviations

ALL: Acute lymphoblastic leukemia
AML: Acute myeloid leukemia
CMML: Chronic myelomonocytic leukemia
CT: Computed tomography
DLBCL: Diffuse large B-cell lymphoma
DVH: Dose-volume histogram
HSCT: Hematopoietic stem cell transplantation
MDS: Myelodysplastic syndrome
MF: Modulation factor
MPAL: Mixed-phenotype acute leukemia
MPN: Myeloproliferative neoplasms
MVCT: Megavoltage computed tomography
OAR: Organs at risk
TBI: Total body irradiation
TMI: Total marrow irradiation
