# Supplementary material for: Total marrow irradiation versus total body irradiation using intensity-modulated helical tomotherapy
Source: J Cancer Res Clin Oncol. 2023 Jan 6;149(9):5965–73. doi: 10.1007/s00432-022-04565-2 (PMC10356893; doi:10.1007/s00432-022-04565-2)
Supplement: Supplementary file 1 — Supplementary file1 (PDF 242 KB) [file 432_2022_4565_MOESM1_ESM.pdf]

Supplemental material

ID	Sex	Age [a] at Admission	Dose [Gy] / Fractions	Primary Diagnosis	Subcalssification	EBMT-Score	Sorrow-Score	Line of Therapy
1	f	14.7	12/6	ALL, Pre-B	no specific mutation	NA	NA	1
2	f	62.7	8/4	ALL, Common-B	BCR-ABL positive	2	2	1
3	f	48.4	12/6	ALL, Common-B	BCR-ABL positive	3	1	1
4	f	40.4	8/4	ALL, Common-B	BCR-ABL positive	3	2	1
5	f	61.6	8/4	AML	NPM1, FLT3-ITD	3	10	1
6	m	44.8	2/1	AML	NPM1, FLT3-ITD, IDH-1, DNMT3A	6	2	2
7	m	55.5	8/4	AML	no specific mutation	5	5	1
8	f	44	12/6	ALL, Common-B	no specific mutation	4	0	2
9	m	15.9	12/6	ALL, Common-B	BCR-ABL positive, Monosomy 7	NA	NA	1
10	m	68.7	8/4	mast cell leukemia	c-KIT	5	3	1
11	f	40.1	8/4	MDS, MPN	no specific mutation	5	8	1
12	f	67.2	2/1	AML	NPM1	5	5	2
13	m	37.2	12/6	ALL, Common-B	BCR-ABL positive	3	3	1
14	m	48.5	8/4	ALL, Pre-T	no specific mutation	3	1	1
15	m	63.9	8/4	ALL, Pre-T	BCR-ABL positive	3	3	1
16	f	50.2	8/4	AML	no specific mutation	6	0	2
17	f	17.8	4/2	AML	no specific mutation	NA	NA	1
18	m	72.2	4/2	AML	NPM1	4	1	2
19	f	49.5	4/2	AML	NPM1, FLT3-ITD	3	5	1
20	f	29.5	8/4	AML	no specific mutation	5	0	2
21	f	69.6	2/1	MDS	DNMT3A, TP53	5	6	1
22	f	57.3	8/4	ALL, Pro-B	MLL-AF4	4	4	1
23	f	51.1	8/4	ALL, Common-B	BCR-ABL positive	3	2	1
24	f	38.6	12/6	ALL, T NOS	BCR-ABL positive	2	0	1
25	f	56.7	8/4	ALL, Pre-T	no specific mutation	4	1	1
26	f	19	12/6	ALCL	ALK	NA	NA	3
27	m	51.7	4/2	AML	NPM1, FLT3-ITD	4	3	1
28	m	49.3	8/4	ALL, Common-B	no specific mutation	5	3	2
29	m	25.4	8/4	AML	no specific mutation	4	2	1
30	f	49.4	8/4	ALL, Common-B	MLL-AF4	3	0	1
31	m	31.1	12/6	MPAL	no specific mutation	3	3	1
32	m	13.4	12/6	ALL, Common-B	no specific mutation	NA	NA	3
33	m	68.9	2/1	AML, CMML	no specific mutation	6	8	1
34	m	65.4	2/1	DLBCL	no specific mutation	6	2	2
35	m	37.9	12/6	Burkitt-ALL	c-myc	4	6	2

Supplemental Table 1: Detailed patient characteristics

(a) TBI

	2 Gy		4 Gy		8 Gy		12 Gy	
	D_{mean}	SD	D_{mean}	SD	D_{mean}	SD	D_{mean}	SD
Eye, l	1.07	0.65	1.95	1.27	2.79	1.91	4.69	2.93
Eye, r	1.06	0.67	1.95	1.30	2.88	1.90	4.68	3.00
Lens, l	0.73	0.78	1.44	1.52	1.71	1.81	2.92	3.22
Lens, r	0.74	0.78	1.40	1.48	1.75	1.82	2.94	3.24
Lung, l	1.79	0.35	3.25	0.69	6.06	0.89	7.79	0.36
Lung, r	1.78	0.37	3.16	0.73	5.96	0.91	7.59	0.46
Kidney, l	1.78	0.39	3.05	0.66	6.33	1.20	7.54	1.89
Kidney, r	1.78	0.39	2.88	0.97	6.15	1.12	7.60	1.86
Spleen	2.04	0.03	4.13	0.05	8.18	0.17	12.19	0.15
Liver	2.06	0.03	4.12	0.05	8.23	0.12	12.33	0.05
Body	2.05	0.03	4.09	0.06	8.13	0.10	12.12	0.22
Skeleton	2.06	0.03	4.13	0.02	8.23	0.09	12.38	0.11

(b) TMI

	2 Gy		4 Gy		8 Gy		12 Gy	
	D_{mean}	SD	D_{mean}	SD	D_{mean}	SD	D_{mean}	SD
Eye, l	0.75	0.16	1.30	0.18	2.62	0.51	4.34	0.85
Eye, r	0.74	0.19	1.21	0.19	2.52	0.49	4.36	0.94
Lens, l	0.50	0.19	0.77	0.12	1.50	0.38	2.59	0.44
Lens, r	0.45	0.15	0.72	0.14	1.49	0.40	2.52	0.41
Lung, l	1.30	0.08	2.49	0.18	5.03	0.26	7.46	0.36
Lung, r	1.27	0.06	2.41	0.14	4.85	0.23	7.31	0.35
Kidney, l	0.68	0.07	1.27	0.19	2.44	0.39	3.97	0.84
Kidney, r	0.62	0.11	1.13	0.21	2.26	0.27	3.58	0.91
Spleen	0.98	0.21	2.19	0.11	4.27	0.27	6.03	0.41
Liver	1.08	0.13	2.18	0.14	4.16	0.25	6.07	0.40
Body	1.48	0.02	2.91	0.05	5.67	0.22	8.61	0.25
Skeleton	2.07	0.01	4.14	0.00	8.27	0.02	12.37	0.06

Supplemental Table 2: Average D_{mean} for (a) TBI and (b) TMI plans with standard deviation (SD) for each organ and Gy subgroup.
